# Retroinverso analogs of spadin display increased antidepressant effects

**DOI:** 10.1007/s00213-014-3683-2

**Published:** 2014-08-02

**Authors:** Julie Veyssiere, Hamid Moha ou Maati, Jean Mazella, Georges Gaudriault, Sébastien Moreno, Catherine Heurteaux, Marc Borsotto

**Affiliations:** 1 Université de Nice Sophia Antipolis, IPMC, 06560 Sophia Antipolis, France; 2 CNRS, IPMC, 06560 Sophia Antipolis, France; 3 Institut de Génomique Fonctionnelle, 141 rue de la Cardonille, 34095 Montpellier Cedex 5, France; 4 Medincell SA, 1 rue Charles Cros, 34830 Jacou, France

**Keywords:** Retro-inverso, Spadin, TREK-1, Depression, Electrophysiology

## Abstract

**Rationale:**

Although depression is the most common mood disorder, only one third of patients are treated with success. Finding new targets, new drugs, and also new drug intake way are the main challenges in the depression field. Several years ago, we identified a new target with the TWIK-related potassium channel-1 (TREK-1) potassium channel, and more recently, we have discovered a peptide of 17 amino acids with antidepressant properties. This peptide, that we called spadin, can be considered as a new concept in antidepressant drug design. Spadin derives from a larger peptide resulting to a posttranslational maturation of sortilin; consequently, spadin can be considered as a natural molecule. Moreover, spadin acts more rapidly than classical antidepressants and does not induce side effects.

**Objectives:**

In this work, we sought analogs of spadin displaying a better affinity on TREK-1 channels and an increased action duration.

**Methods:**

Analogs were characterized by electrophysiology measurements, by behavioral tests, and by their ability to induce neurogenesis.

**Results:**

We identified two retro-inverso peptides that have kept the antidepressant properties of spadin; particularly, they increased the hippocampal neurogenesis after a 4-day treatment. As spadin, these analogs did not induce side effects on either pain, epilepsy processes, or at the cardiac level.

**Conclusions:**

Together, our results indicated that spadin retro-inverso peptides could represent new potent antidepressant drugs. As exemplified by spadin in the field of depression, retro-inverso strategies could represent a useful technique for developing new classes of drugs in a number of pathologies.

## Introduction

Depression is a devastating neuropsychiatric disorder and affects approximately 20 % of the population. Depression is predicted to be a major cause of morbidity worldwide in the next 10 years and will induce an important economic burden (Greenberg et al. 2003; Moussavi et al. 2007). Depression is a multifactorial and multigenic disease characterized by many symptoms like fatigue, anhedonia, pessimism, irritability, sleep troubles, increased or decreased appetite, guiltiness, and suicidal tendencies (Nestler et al. 2002). Sixty years ago, antidepressant treatments have been revolutionized by the discovery of tricyclic antidepressants and monoamine oxidase inhibitors. Later, a second generation of antidepressants was developed with the selective serotonin or norepinephrine selective reuptake inhibitors. Despite their efficacy, around one third of patients remain unresponsive to these drugs. Moreover, they display some adverse side effects and have a long onset of action (Sicouri and Antzelevitch 2008). Consequently, it was necessary to develop new antidepressant molecules with new pharmacological targets.

We previously demonstrated that the inhibition of TREK-1 led to an antidepressant phenotype (Heurteaux et al. 2006b). Our researches led to the identification of a specific inhibitor of TREK-1 channel called spadin (Mazella et al. 2010). Spadin resulted from modification of the sortilin receptor (Mazella et al. 1998). Spadin is a 17 amino acid peptide which was designed from a 44 amino acid peptide (called PE) released by furin in the late Golgi apparatus during the posttranslational maturation of the sortilin receptor (Munck Petersen et al. 1999). Spadin is able to block the TREK-1 potassium channel current and displays antidepressant effects in different behavioral tests (Mazella et al. 2010). Additionally, like other antidepressant drugs, spadin is able to increase neurogenesis and serotoninergic transmission. More interestingly, unlike the most used antidepressants, which need 21 days to be efficient, spadin has a quicker onset of action since it is able to induce these improvements only after a 4-day treatment (Mazella et al. 2010). In the two pore (K_2P_) potassium channel family, spadin is specific for TREK-1 channels (Moha ou Maati et al. 2011b). Moreover, the activation of TREK-1 channels was demonstrated to be beneficial in different functions such as general anesthesia, neuroprotection by the way of polyunsaturated fatty acids, pain, ischemia, and epilepsy (Alloui et al. 2006; Heurteaux et al. 2006a; Lauritzen et al. 2000; Noel et al. 2009). Nevertheless, blockade of TREK-1 channels by spadin does not interfere with these functions. In other words, spadin is devoid of side effects on TREK-1-controlled functions (Moha ou Maati et al. 2011b). Importantly, spadin does not induce any cardiac dysfunctions, and both systolic pressure and pulses are not affected by a 3-week spadin treatment. Additionally, spadin is unable to block the two most important repolarizing currents in the heart (I_KR_, I_KS_) (Moha ou Maati et al. 2011b). Taken together, these properties are strong evidences for considering spadin as an antidepressant drug of a new generation.

With the aim to identify new analogs displaying a better efficacy than spadin, we synthesized different portions of human sortilin either in natural L-configuration or retro-inverso configuration. This approach consists in synthesizing peptides in which not only the chirality of amino acid is inverted by replacing all l-amino acids by d-amino acids but also the amino acid sequence is reversed (Bonny et al. 2001; Chorev and Goodman 1995). In such a way, the side chains of amino acids are in a similar position to that of the native peptide (Bonny et al. 2001; Chorev and Goodman 1995; Van Regenmortel and Muller 1998). Very often, retro-inverso peptide properties are the same or close, sometimes better, than the parent l-peptides and, overall, retro-inverso peptides are more resistant to proteolysis (Taylor et al. 2010; Weeden et al. 2011).

We first screened 12 spadin analogs for their ability to block TREK-1 channel activity. The two most efficient were retained for further studies using behavioral tests and measurements of their effects on neurogenesis. Because the TREK-1 channel deletion was shown to be deleterious for epilepsy or pain (Alloui et al. 2006; Heurteaux et al. 2004; Noel et al. 2009), we studied the effects of analog treatments on these potential side effects. We also checked the analog harmlessness on the two main cardiac repolarizing currents I_KR_ and I_KS_ that are essential in cardiac function.

## Materials and methods

### Cell culture

The human-TREK-1/HEK293 cell line (h-TREK-1/HEK) (Moha ou Maati et al. 2011a) and HEK-IKS cell line (Ducroq et al. 2010) were grown in the presence of 0.5 mg/mL G418 in Dulbecco’s modified Eagle’s medium supplemented with 10 % (*v*/*v*) heat-inactivated fetal bovine serum containing 1 % (*v*/*v*) penicillin/streptomycin in an atmosphere of 95 % air/5 % CO_2_ as previously described (Moha ou Maati et al. 2011a).

HEK-293 native cells were grown in serum in an atmosphere of 95 % air/5 % CO_2_ in Dulbecco’s modified Eagle’s medium supplemented with 10 % (*v*/*v*) heat-inactivated fetal bovine containing 1 % (*v*/*v*) of penicillin/ streptomycin and Glutamax X 1. Cells were plated at a density of 20,000 cells/35 mm dish, and after 24 h, cells were transfected using the Jet PEI method (Polyplus, France) with 25 ng/35 mm dish of p-IRES-HERG channel vector. Patch clamp experiments were carried out 48 h after transfection.

### Electrophysiology

All electrophysiological experiments were performed on h-TREK-1/HEK cells seeded at a density of 20,000 cells/35 mm dish after 2–6 days of culture. All electrophysiological recordings were performed in whole cell configuration of the patch clamp technique except for I_KS_ measures which were obtained by using the patch clamp perforated configuration (amphotericin B 0.9 mg/mL in the pipette medium) (Moha ou Maati et al. 2011b). Each current was evaluated by using a RK 400 patch clamp amplifier (Axon Instrument, USA), low-pass filtered at 3 kHz, and digitized at 10 kHz using a 12-bit analog-to-digital converter digidata (1322 series, Axon Instrument, USA). All current amplitudes are expressed in current densities. Results are expressed as mean ± standard error of the mean (SEM). Patch clamp pipettes were pulled using vertical puller (PC-10, Narishige) from borosilicate glass capillaries and had a resistance of 3–5 MΩ. The bath solution contained (in mM) 150 NaCl, 5 KCl, 3 MgCl_2_, 1 CaCl_2_, and 10 4-(2-hydroxyethyl)piperazine 1- ethane sulfonic acid (HEPES) adjusted to pH 7.4 with NaOH. The pipette solution contained (in mM) 155 KCl, 3 MgCl_2_, 5 EGTA, and 10 HEPES adjusted to pH 7.2 with KOH. TREK-1 currents were evaluated at room temperature (21–22 °C) in the presence of a cocktail of potassium channel inhibitors (K^+^ blockers, 3 mM 4-aminopyridine (4-AP), 10 mM tetraethylammonium (TEA), 10 μM glibenclamide, 100 nM apamin, and 50 nM charybdotoxin). Stimulation protocols and data acquisition were carried out using a microcomputer (Dell Pentium) with a commercial software and hardware (pClamp 8.2). Currents were recorded by voltage clamp steps to membrane potentials of −100 to +60 mV in 20-mV steps applied from a holding potential of −80 mV. The duration of depolarization pulses was 825 ms, and the pulse cycling rate was 5 s. TREK-1 current amplitudes were evaluated at the end of stimulation pulses. Cells were continuously superfused with microperfusion system. TREK-1 inhibitory effects of spadin or analogs were performed on arachidonic acid pre-activated currents. Spadin and analogs were tested at the unique dose of 100 nM on TREK-1 channel activity and at 10 μM on I_KR_ and I_KS_ currents. For both analogs 3 and 8, TREK-1 concentration-dependent inhibitions were performed by applying concentrations ranging between 1 nM and 1 μM.

I_KS_ currents were activated by voltage clamp steps of membrane potentials from −100 to +100 mV in 20-mV steps applied from a holding potential of −80 mV. Tail currents were generated by repolarization to −40 mV. Duration of both depolarization and repolarization pulses was 2.4 s, and the pulse cycling rate was 10 s. I_KR_ currents were activated by voltage clamp steps of membrane potentials from −100 to +100 mV in 10-mV steps applied from a holding potential of +80 mV, and tail currents were generated by a repolarization to +40 mV. The duration of both depolarization and repolarization pulses was 1 s, and the pulse cycling rate was 5 s. The amplitudes of I_KS_ and I_KR_ currents were calculated at both the end of the first pulse and the peak of the tail pulse.

### Animals

Naïve male C57Bl/6J mice from 7 to 9 weeks old were used in all experiments (Janvier Laboratory, Saint Berthevin, France). Mice were housed (10 animals per cage) under a 12:12 light–dark cycle (light on at 8:00 am) in a ventilated room at a temperature of 22 ± 1 °C. Animals had free access to water and food (A03; SAFE, Augy, France). All experiments were conducted according to policies on the care and use of laboratory animals of the Society for Neuroscience and also with respect to national laws on animal use. The local ethics committee (CIEPAL) approved the experimental protocols (authorization number 00736–02).

### Treatments

**Fig. 1:**
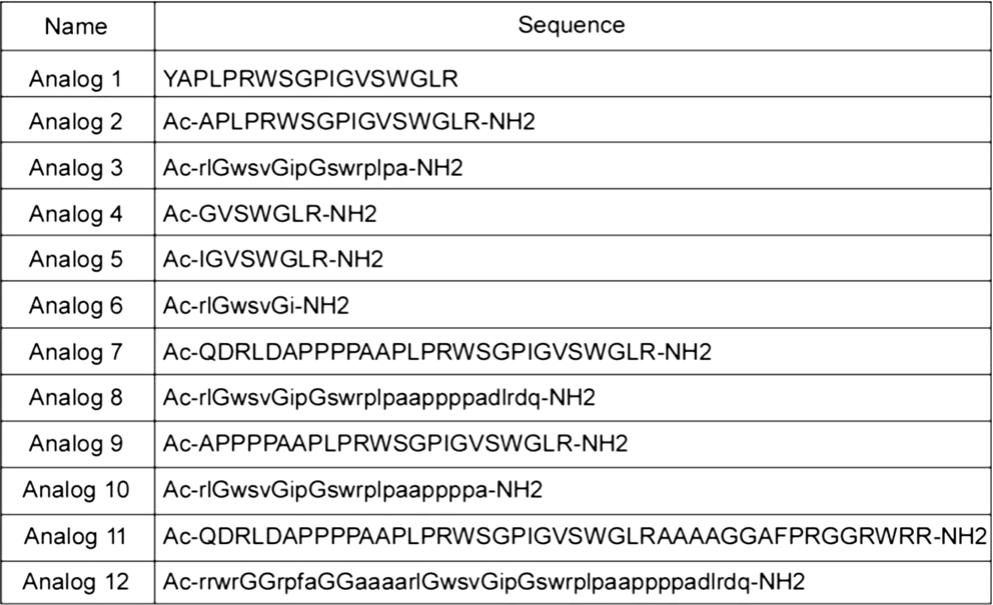
Sequences of spadin analogs. Peptide sequences are presented using the *one-letter* nomenclature. Amino acids in L-configuration are shown in *capital letters*, while amino acids in D-configuration are shown as *lowercase letters. Ac* corresponds to acetyl group, *−NH2* to amide group, and spadin and PE correspond to sequences 1 and 11, respectively

Spadin was synthesized by Gencust (France). Other peptides (see Fig. 1) were synthesized by the American Peptide Company (Sunnyvale, CA, USA). Peptides were purified by the supplier, purity >80 %. The purity was verified by analytical high-performance liquid chromatography (HPLC) and mass spectral analysis.

Stock solutions were prepared at 10^−3^ M in distilled water, and before injection, spadin or analog solutions were diluted in NaCl 0.9 % to obtain the different concentrations used for treatments. Corticosterone (Sigma-Aldrich, France) was dissolved in drinking water at the concentration of 3.5 mg/L in the presence of 4.5 g/L of beta-cyclodextrin. The mixture was filled into opaque bottles to protect from the light and mice had a free access to this solution. Fluoxetine (Sigma-Aldrich, France) was dissolved in drinking water at the dose of 80 mg/L and administered during 21 days. For i.p. administration, fluoxetine (TEVA Santé, France) was dissolved in NaCl 0.9 % at a concentration of 0.75 mg/mL. The total amount injected was adjusted to obtain 3 mg/kg. Spadin and analogs were administered by intravenous (i.v.) injection. For acute treatment, drugs were administered in a single 100-μL bolus 30 min prior to the beginning of the behavioral tests. For subchronic treatment, drugs were injected during four consecutive days, and behavioral tests were performed on day 5, without additional injection.

### Behavioral tests

Behavioral experiments were performed with naïve mice. The experimenter was blind to experimental groups. All mice were naïve to every behavioral test used.

#### Forced swimming test (FST) (Porsolt et al. 1977)

The animals were individually placed in a non-escapable cylinder (height 30 cm, diameter 15 cm) filled with 15-cm water at 22 ± 1 °C. The trial was conducted for 6 min. The total period of immobility was manually measured during the last 4 min of the test. A mouse was considered immobile when it remained floating with only slight movements to keep its head above water.

#### Novelty suppressed feeding (NSF) (Santarelli et al. 2003)

The NSF paradigm is a 2-day test protocol. On day 1, mice were deprived from food. On day 2, mice were placed in a highly brightly lit area, in a plastic box (45 × 45 × 20 cm), with a floor covered with wooden bedding. The test was carried out during a 10-min period. During this time, the latency to eat was measured. During the test, a single pellet of food was placed in the center of the box, on a white platform.

#### Learned helplessness (LH) (Caldarone et al. 2000)

The learned helplessness test is divided in a 4-day training session and 1-day test session.

During the training session, mice were exposed to 360 inescapable 2-s footshocks, with an intertrial interval of 8 s.

The test consists in 30 trials separated by a 30-s interval. One trial was defined as a 5-s period before shock onset and was terminated when the mouse moved to the second compartment or at the end of the shock onset. During the test, the latency to escape for each mouse during every trial was recorded.

#### Tail immersion test (Alloui et al. 2006)

Mice were i.v. injected with 10 μg/kg of spadin in a bolus of 100 or 100 μL of a saline solution (0.9 % NaCl) 30 min before the beginning of the test. The tail was immersed in a water bath at 48 °C until withdrawal was observed (cutoff time 30 s). Two separate withdrawal latency time determinations were averaged (Alloui et al. 2006).

#### Seizure induced by kaïnate (Tsirka et al. 1995)

Kaïnate solutions were prepared in a solution of 140 mM NaCl (saline solution).

Spadin 10 μg/kg or vehicle was i.v. injected and, immediately after the injection, kaïnate, 25 mg/kg, was i.p injected in a bolus of 100 μL. Mice (*n* = 10 per group) were monitored during 2 h for onset and extent of seizures. Six levels of seizure severity were defined: (1) immobility, (2) head/neck movements, (3) clonic unilateral activity, (4) clonic bilateral activity, (5) generalized convulsions, and (6) death. Seizure severity was blindly scored (Tsirka et al. 1995). The seizure index was calculated by averaging the points for seizure activity in each group (*n* = 10 per treatment).

### Mouse locomotor activity

To determine whether analog 3 induced a change in locomotor activity, mice (*n* = 8 per group) were injected with the saline solution or analog 3 (10^−5^ M in 100 μL bolus, i.v.) 30 min before starting the test session. Locomotor activity was monitored individually for 24 h using an infrared photobeam activity monitoring system (Imetronic, Pessac, France), which measured consecutive horizontal beam breaks. Testing was in transparent plastic cages (43 × 20 × 20 cm^3^) with fresh bedding in a grid of 8 cm horizontal infrared beams. Locomotor activity was defined as breaking of consecutive photobeams. Movements were recorded and totalized for each 10-min time section. Six periods were pooled to obtain data for 1 h of time. Different movements were monitored: the coming-and-going between the back and the front of the cage, climbing, and other movements in the back or the front of the cage. Mice were kept under standard laboratory conditions: 12:12 light–dark cycle with free access to food and water during the experiment. Data are the mean value of eight animals per condition, and bars represent SEM.

### Spadin analog recovery in the brain after i.v. injection

Prior to injection, C57BL/6J males were warmed for 5–10 min with an overhead heat lamp to dilate the veins. Then, they were placed in a constrained box and injected in the caudal vein with 100 μL of either 100 μM spadin analog 3 or 0.9 % NaCl solution. The brain was removed either immediately or 30 and 60 min after injection, and the peptide content was recovered by acidic extraction and analyzed by HPLC using a Jasco apparatus equipped with an analytic RP18 Lichrosorb column as previously described (Checler et al. 1986). Elutions of HPLC products were carried out by means of a 50-min linear gradient of acetonitrile from 10 to 60 % at a flow rate of 1 mL/min. Under these conditions, the analog was eluted at 31.5 min. The analog recovered from the brain and identified by mass spectrometry was quantified using a standard curve made with increasing concentrations of analog 3 from 50 to 200 pmol.

### Neurogenesis

One day after 5-bromo-2′-deoxyuridine (BrdU) injections, 12 mg per animal divided in four bolus of 300 μL injected every 2 h, mice were anesthetized with isoflurane and transcardially perfused with 20 mL of NaCl 0.9 % followed by 20 mL paraformaldehyde 4 %/NaCl 0.9 %. By using a vibratome (Leica), brains were cut into 40-μm sections, throughout the entire hippocampus. Eight slices, from bregma 3.3 to bregma 5.3, were retained to process the BrdU immunohistochemistry as previously described (Heurteaux et al. 2006b). For each BrdU labeling, slices were first incubated with a mouse monoclonal anti-BrdU antibody (1/8,000, Becton Dickinson). For chromogenic immunodetection, sections were incubated during 2 h in biotin-conjugated species-specific secondary antibodies (1/400; Vector laboratories) followed by a peroxidase-avidin complex solution, to amplify the reaction. The peroxidase activity of immune complex was visualized with DAB staining using the VectaStain ABC kit according to the manufacturer’s protocol (Vector Laboratories).

### Statistics

Data were expressed as mean ± SEM. Statistical analysis of differences between groups was performed by using Mann-Whitney test. In all analyses, the level of significance was set at *p* < 0.05 (*), *p* < 0.01 (**), and *p* < 0.001 (***).

In the learned helplessness test, latencies to escape were recorded for each of the 30 trials. The average value was calculated for each of the five trials; thus, six blocks of values were obtained in addition to the overall average escape latency. A Mann-Whitney test was carried out on both overall latencies and blocks of trials.

## Results

### Electrophysiological characterization of spadin’s analog on TREK-1 channel affinity

**Fig. 2:**
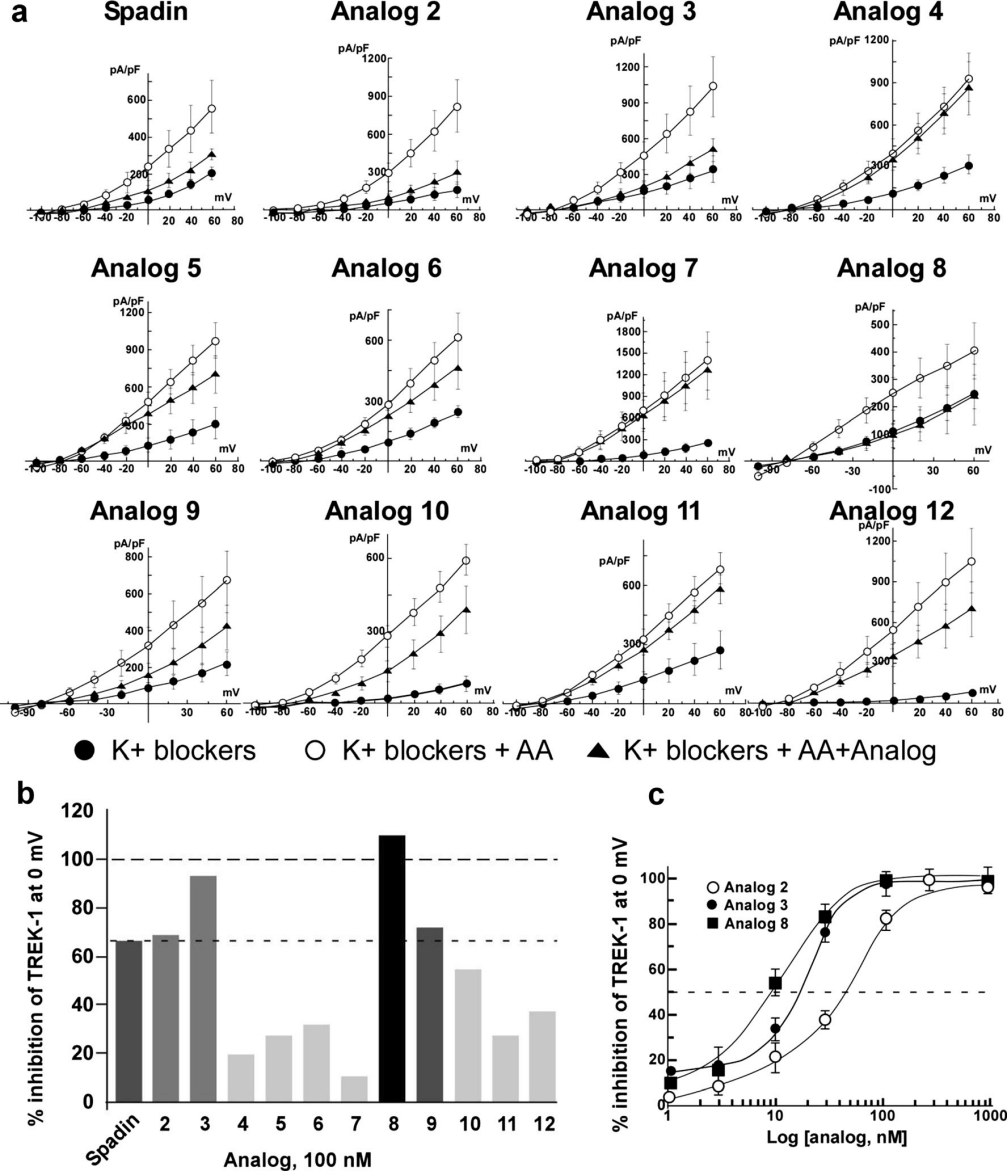
*I*–*V* curves of spadin and its analogs. All experiments were performed on h-TREK-1/HEK cell line in the presence of a mixture of K^+^ channels blockers and by using the whole cell configuration of the patch clamp technique. **a** Control currents (*black-filled circles*, K^+^ blockers) were amplified by the application of 10 μM of arachidonic acid (*white-filled circles*, K^+^ blockers + AA). After application of 100 nM of spadin or its analogs, remaining currents were measured (*black-filled triangles*, K^+^ blockers + AA + spadin or analog). **b** Percentage of inhibition of the TREK-1 current measured at 0 mV obtained by application of 100 nM of spadin and different analogs. **c** Dose–response curves obtained by measuring the percentage of TREK-1 current inhibition at 0 mV with analog 2 (*white-filled circles*), analog 3 (*black-filled circles*), and analog 8 (*black-filled squares*)

In order to identify analogs having a better affinity than spadin for TREK-1 channels, we first studied their ability to block the channel activity in the h-TREK-1/HEK cell line (Moha ou Maati et al. 2011a). TREK-1 channels expressed in this cell line have conserved all their modulating properties (Moha ou Maati et al. 2011a). By using the whole cell configuration of the patch clamp technique, analog 2 to analog 12 (Fig. 1) were tested at 100 nM (*n* = 10 to 12) and the analog 1 corresponding to spadin (Mazella et al. 2010; Moha ou Maati et al. 2011a, b) was used as reference. Our data indicated that only two analogs, analogs 3 and 8, presented an increased blockade effect when compared to spadin (Fig. 2a, b). Analog 2 that corresponds to the N-terminal-acetylated and C-terminal amidated form of spadin displayed similar activity to spadin. IC_50_ values calculated from dose–response curves were of 11.5 ± 0.59 and 9.95 ± 0.85 nM for analogs 3 and 8, respectively (Fig. 2c). These values had to be compared to 56.39 ± 0.01 nM determined for spadin on the same cell line (Moha ou Maati et al. 2011a), noting that analog 2 had an IC_50_ of 60 ± 0.41 nM (Fig. 2c). These data indicated that both analogs 3 and 8 have a sixfold higher affinity for TREK-1 channels. We retained these analogs in order to investigate their potential antidepressant properties.

### Antidepressant effect of spadin’s analogs after an acute treatment

**Fig. 3:**
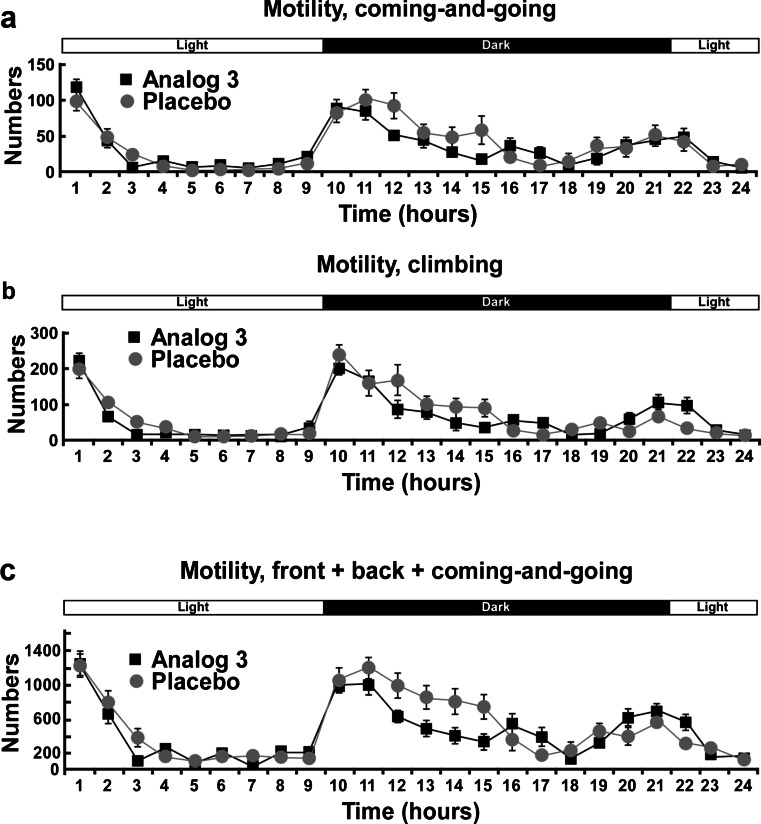
Spontaneous locomotor activity of analog 3-injected mice. Mice were injected 30 min before to be placed in an infrared photobeam activity monitoring system. Spontaneous locomotor activity was monitored individually for 24 consecutive hours. The number of coming-and-going (**a**), climbing (**b**), and total movements except climbing (**c**) were monitored for each mouse for 10 min section and pooled by 6 to obtain values corresponding to 1 h. Light and dark periods are indicated by the *bar above profiles*

Because the FST is based on the immobility and influenced by molecules that spontaneously increase the general activity, we controlled the effect of analogs on mouse locomotion. By using an infrared photobeam activity monitoring system, we showed that there was no significant difference in locomotor activities between analog 3- and saline-treated mice within 24 h after the drug injection (Fig. 3). Coming-and-going (Fig. 3a), climbing (Fig. 3b), and total movements (Fig. 3c) were very similar in both conditions. These results indicate that the difference in the immobility time we further observed in the FST was really due to the effect of the analog treatment and not to a change in the locomotor activity.

**Fig. 4:**
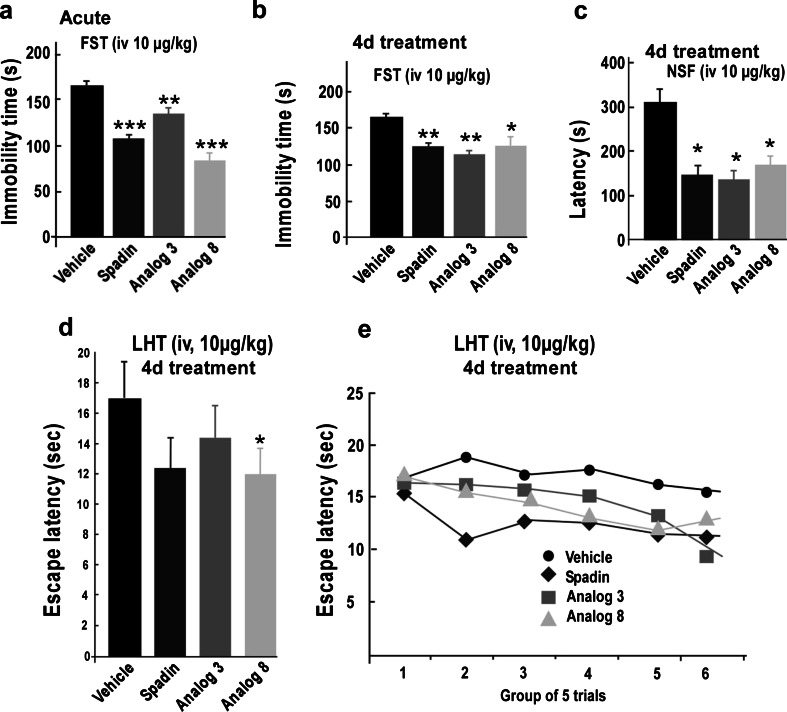
Behavioral tests with spadin and analogs 3 and 8. **a** FST performed after an acute treatment, immobility times were measured 30 min after the i.v. injection of drugs, 10 μg/kg in a single bolus of 100 μL of NaCl 0.9 %. **b** FST performed after a subchronic treatment (4 days, *4d*); immobility times were measured on the fifth day after a daily i.v. injection of drugs, 10 μg/kg in a single bolus of 100 μL of NaCl 0.9 % for four consecutive days. **c** NSF performed after a subchronic treatment (4 days, *4d*); latencies to feed were measured on the fifth day after a daily i.v. injection of drugs, 10 μg/kg in a single bolus of 100 μL of NaCl 0.9 % for 4 days. **d**, **e** LHT performed after a subchronic treatment (4 days, *4d*); latencies to feed were measured on the fifth day after a daily i.v. injection of drugs, 10 μg/kg in a single bolus of 100 μL of NaCl 0.9 % for 4 days. **d** Mean escape latencies for the entire experiment. **e** The mean escape latencies by blocks of five trials. *p < 0.05, **p < 0.01, ***p < 0.001

The antidepressant effects of both analogs were first studied in the FST after an acute injection (Mazella et al. 2010). Here again, spadin was used as control. A 10-μg/kg acute i.v. injection of spadin or both analogs 3 and 8 significantly reduced the immobility time of mice compared to saline-injected mice (Fig. 4a). Values were 166.13 ± 5.54, 107.40 ± 5.05, 135.10 ± 8.11, and 83.60 ± 9.01 s for saline, spadin (*U* = 0, *p* < 0.001), analog 3 (*U* = 8, *p* = 0.01), and analog 8 (*U* = 0, *p* = 0.001), respectively (*n* = 10 for each group).

### Antidepressant effect of spadin’s analogs after a subchronic treatment

The main goal of this study was to find a molecule that can be used in clinic. Thus, we needed a molecule that remained active after several days of administration. Consequently, as already performed with spadin (Mazella et al. 2010), we pursued our study after a subchronic administration of both analogs.

In the FST, subchronic treatments of 4 days (10 μg/kg i.v. injected once a day) with spadin or analogs induced a significant decrease of immobility times. Immobility times observed were of 161.80 ± 8.12 s, 123.70 ± 7.16 s (*U* = 10.5, *p* < 0.01), 114.9 ± 9.82 s (*U* = 10.5, *p* < 0.01), and 124.1 ± 10.53 s (*U* = 17.5, *p* < 0.05) for saline solution, spadin, analog 3, and analog 8, respectively (Fig. 4b).

Similar results were obtained in the novelty suppressed feeding test. Spadin and both analogs reduced the latency to feed. Values were of 305.00 ± 62.47 s, 151.11 ± 17.70 s (*U* = 13, *p* < 0.05), 143.88 ± 23.42 s (*U* = 11, *p* < 0.05), and 167.00 ± 22.96 s (*U* = 13, *p* < 0.05) for saline solution, spadin, analog 3, and analog 8, respectively (Fig. 4c). Although weaker, this antidepressant effect was also observed with learned helplessness test (LHT) (Fig. 4d, e).

Our data clearly indicated that, as spadin, analogs are efficient after only 4 days of treatment.

### Analog stability

**Fig. 5:**
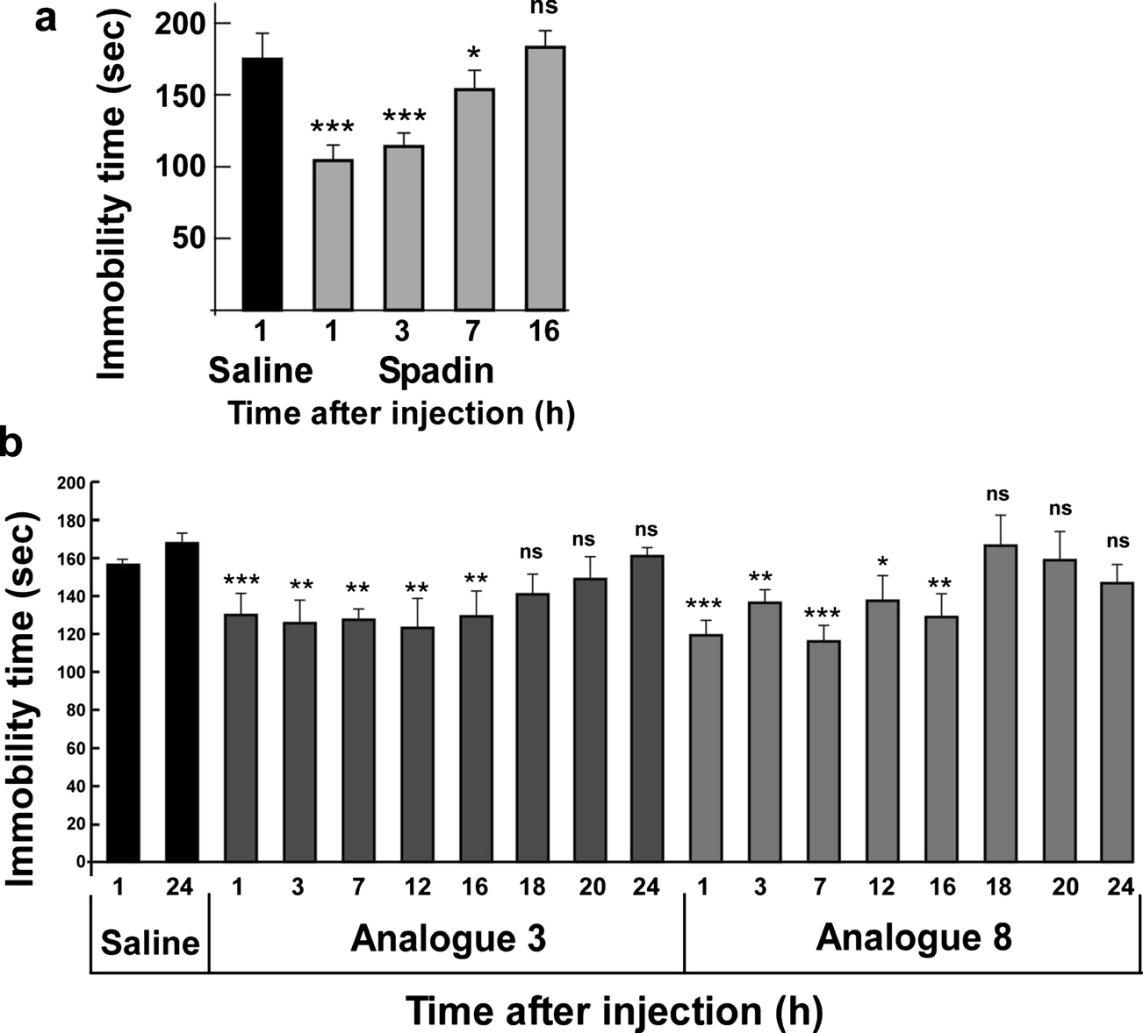
In vivo stability of spadin and analogs 3 and 8. Using FST, we compared the in vivo action duration of spadin (**a**) with both analogs 3 and 8 (**b**). For each drug at each, times animals were naïve. *p < 0.05, **p < 0.01, ***p < 0.001, *ns* nonspecific

For improving the spadin efficacy, in addition to an increased affinity, analogs have to be more stable when injected in vivo. Measured with the FST, the efficacy of spadin decreased from 100 % at *t* = 1 h after the injection to 0 % at *t* = 16 h, with intermediate values of 84 % at *t* = 3 h and 30 % at *t* = 7 h (Fig. 5a). Times of immobility were of 170.3 ± 4.5 s, 102.4 ± 6.2 s (*U* = 0, *p* < 0.001), 113.2 ± 5.0 s (*U* = 0, *p* < 0.001), 150.8 ± 6.5 s (*U* = 19, *p* < 0.05), and 175.3 ± 7.5 s (Fig. 5a). These data indicated that the biological half-life time of spadin is around 6 h.

Then, analogs 3 and 8 were tested at different times, 1, 3, 7, 12, 16, 18, 20, and 24 h, after the injection (*n* = 10 naïve mice at each time). Saline-injected animals were only tested at 1 and 24 h (Fig. 5b). It appeared that both analogs remained efficient to reduce the immobility time after 16 h. The immobility times were very similar between 1 and 16 h, 123.4 ± 7.0 and 129.6 ± 12.7 s, and 121.7 ± 5.2 and 129.1 ± 12.0 s for analog 3 and analog 8, respectively (Fig. 5b). The mean value for saline-treated animals was of 162.7 ± 4.7 s (Fig. 5b).

**Fig. 6:**
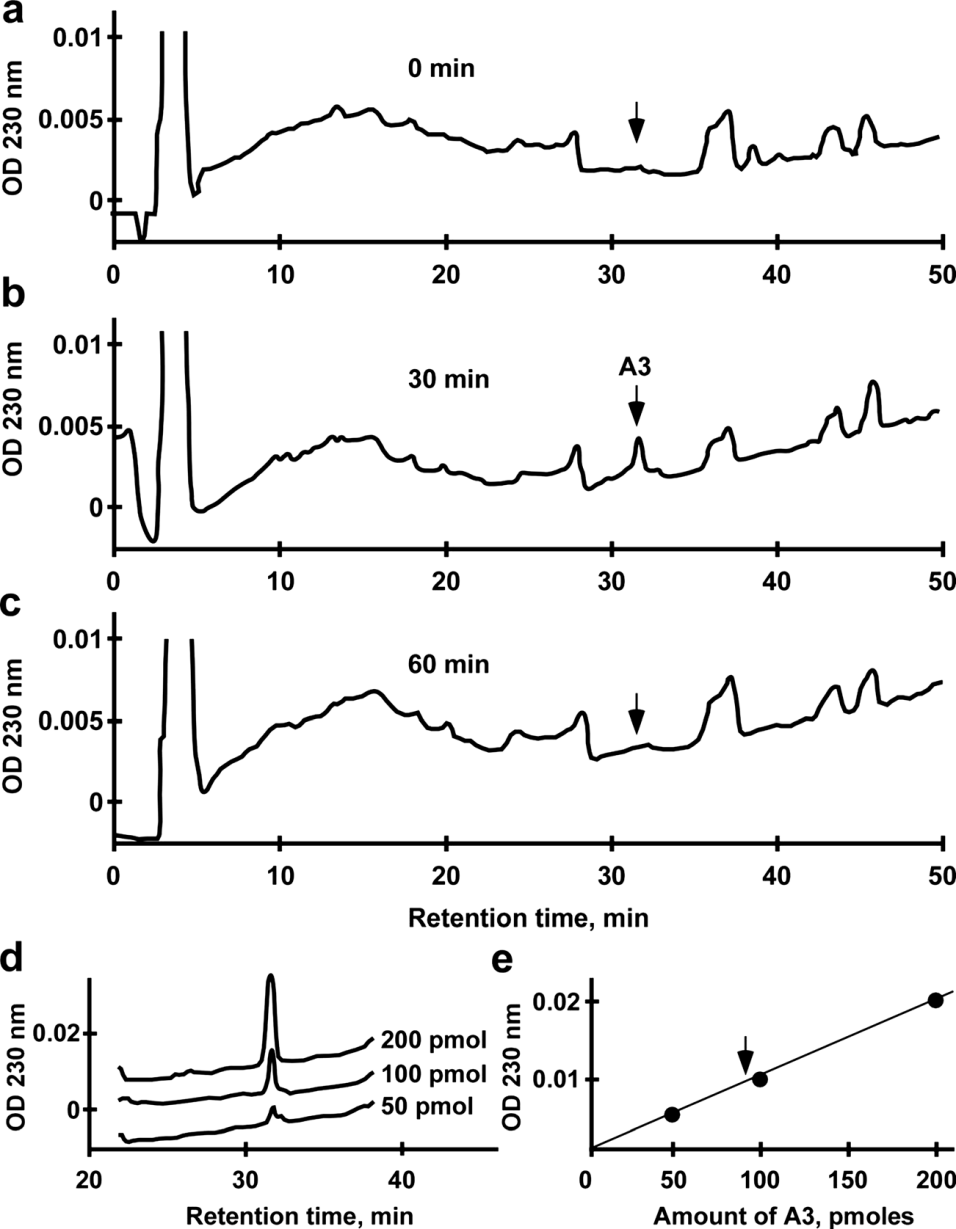
Analog 3 crosses the blood–brain-barrier. **a**–**c** HPLC profiles of analog 3 recovered in the brain at 0 min (**a**), 30 min (**b**), and 60 min (**c**) after i.v. injection. The brain was subjected to acidic extraction, and the extracted peptide content was analyzed by reverse-phase HPLC. The retention time for analog 3 is indicated by the *arrow* (31.5 min). **d**, **e** 50, 100, and 200 pmol of analog 3 were directly quantified by HPLC (**d**) and the linear representation of the OD obtained as a function of the amount of peptide (**e**) allowed us to determine the amount of analog 3 recovered in the brain extract (*arrow*)

In the aim to determine the ability of analogs to cross the blood–brain-barrier, we used analog 3 as the model. Of analog 3, 10 nmol was intravenously injected, and the amount recovered in the brain was estimated by HPLC analysis (Fig. 6). In the peptide content analyzed from a half brain extract after 30 min of injection, a peak not present in the basal condition (0 min; Fig. 6a) was observed with a retention time of 31.5 min (Fig. 6b). This peak disappeared after 60 min (Fig. 6c). This peak was identified as analog 3 by its retention time identical to standards directly analyzed by HPLC (Fig. 6d) and by mass spectrometry. From different amounts detected by HPLC (Fig. 6d), we determined the amount of analog 3 recovered in the brain (Fig. 6e, arrow) which was estimated to be 100 pmol for a half brain then to be 200 pmol per brain. Therefore, we can estimate to 2 % the yield of analog 3 to cross the blood–brain-barrier. This value corresponds to an increase by a factor 20 of the percentage estimated for spadin (Mazella et al. 2010).

These data clearly indicated that both analogs have better in vivo action duration than spadin itself.

### Effects of analogs on neurogenesis

**Fig. 7:**
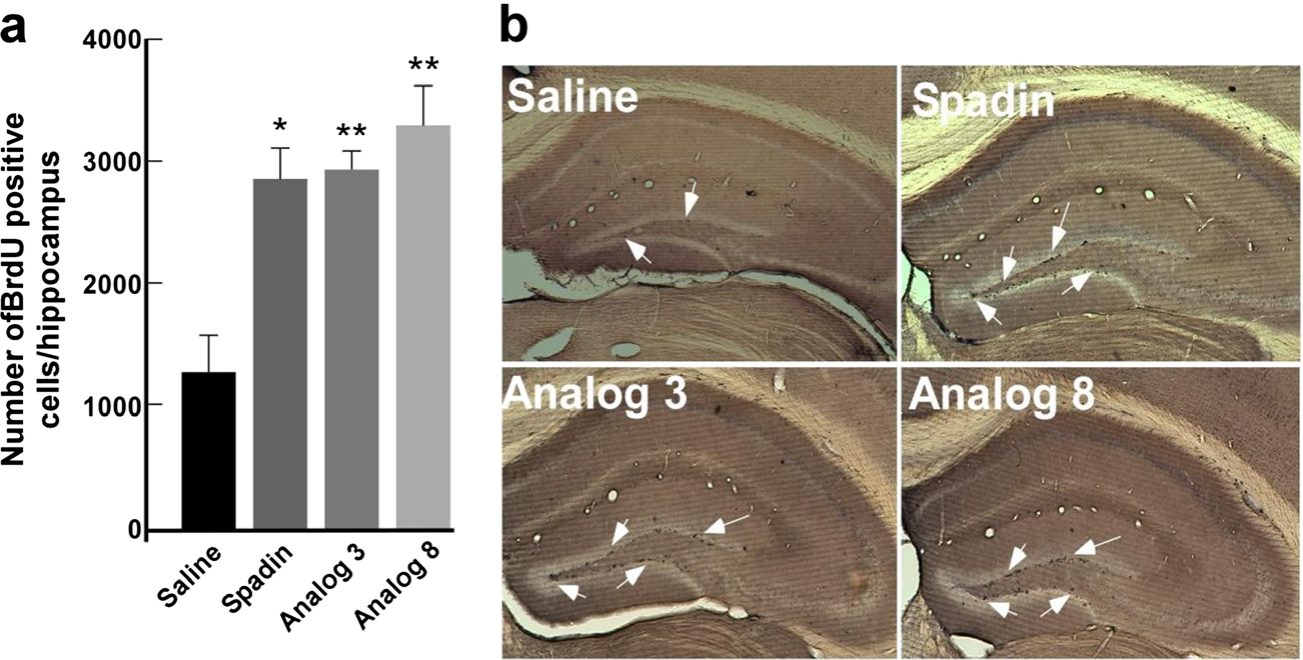
Neurogenesis. **a** Representative photomicrographs of BrdU-labeled neurons in the dentate gyrus of the mouse hippocampus treated for 4 days either with saline, spadin, analog 3, or analog 8 (i.v., 10 μg/kg for all drugs). *Arrows* showed examples of positive cells. **b** Quantitation of BrdU-positive cells of hippocampus treated with saline, spadin, analog 3, or analog 8 for four consecutive days by an i.v. injection of drugs at 10 μg/kg in a single bolus of 100 μL of NaCl 0.9 %. *p < 0.05, **p < 0.01

It was previously shown that a 4-day subchronic treatment with spadin increased the hippocampal neurogenesis (Mazella et al. 2010). We investigated the ability of both analogs to induce a neoneurogenesis in the subgranular zone (SGZ) of the hippocampal dentate gyrus, by counting the number of progenitor cells that incorporated the DNA synthesis marker BrdU. In SGZ, a 4-day treatment with spadin or analogs significantly increased the number of BrdU-positive cells by at least a factor 2 when compared to saline conditions (Fig. 7a, b).

These data indicated that both analogs are able to induce neurogenesis.

### Potential side effects on -TREK-1controlled functions

**Fig. 8:**
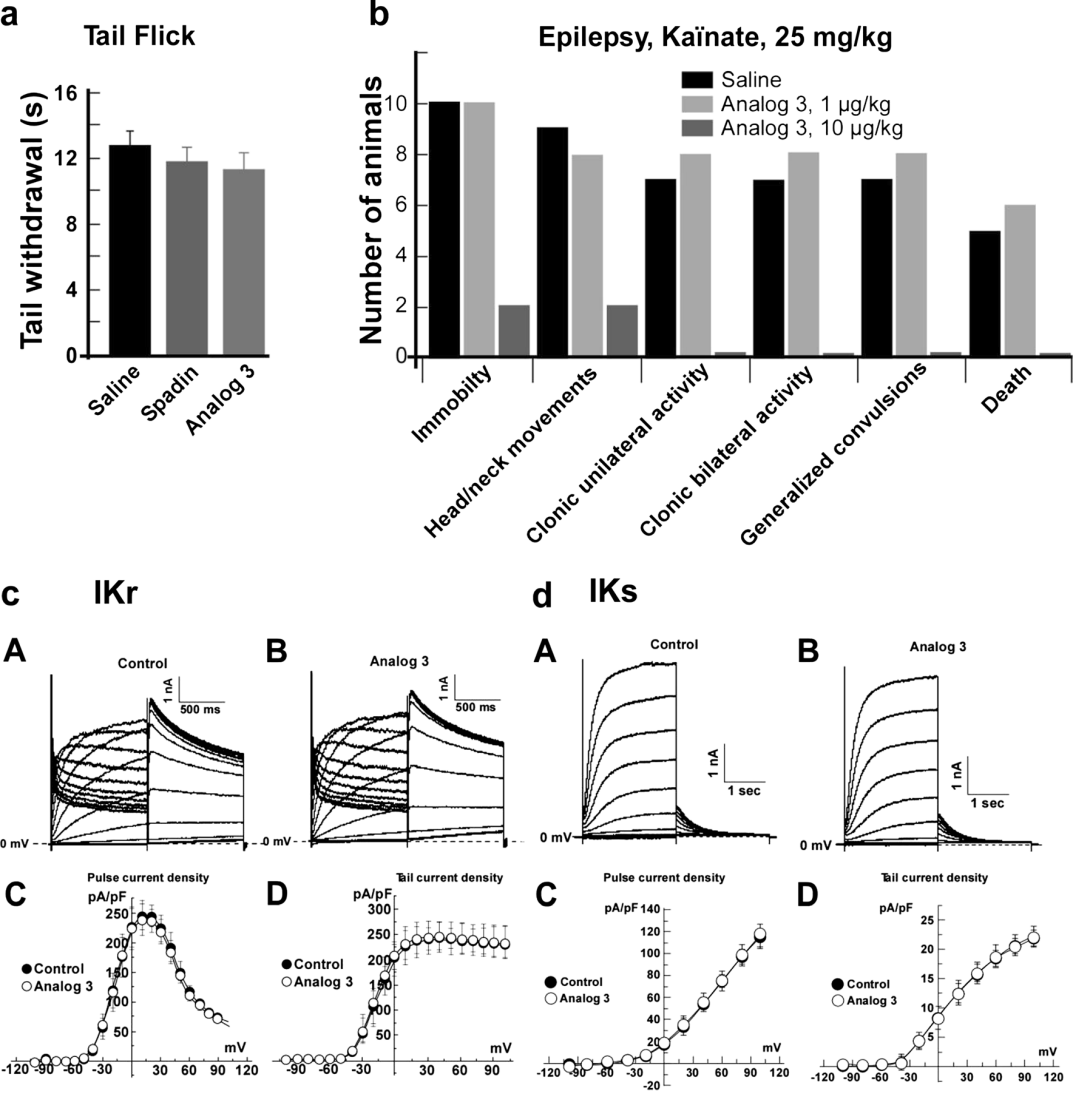
Side effects. **a** Tail flick test (*n* = 10 per group). For each mouse, the time to withdraw its tail immersed in a water bath at 48 °C was measured twice and averaged. There was no significant difference between saline, spadin, or analog 3-treated mice. **b** Epilepsy. Seizures were triggered by an i.p. injection of kaïnate (25 mg/kg) that was immediately followed an i.v. injection of saline solution or analog 3  at 1 or 10 μg/kg in 100 μL bolus (*n* = 10 per group). The number of animals reaching the different levels of severity was counted. **c**, **d** Analog 3 (10 μM) effects on the cardiac delayed K^+^ rectifying currents I_KR_ and I_KS_. **c** Typical traces of human whole cell hERG current recordings in the absence (control) (**a**) or in the presence of 10 μM analog 3 (**b**). **c**, **d** *I*/*V* curves obtained with the first pulse (**c**, end of pulse) and the second pulse (**d**, tail current) of hERG current (*n* = 5). **d** Typical traces of human whole cell human-I_KS_ current recordings in the absence (control) (**a**) or in the presence of 10 μM analog 3 (**b**). **c**, **d** *I*/*V* curves obtained with the first pulse (**c**, end of pulse) and the second pulse (**d**, tail current) of human-I_KS_ current (*n* = 5)

Because TREK-1 channels are being involved in pain, we analyzed the effects of both analogs 3 and 8 on thermal pain by using the tail immersion test. It clearly appeared that both analogs as well as spadin did not increase the thermal pain sensation (Fig. 8a). Measured tail withdrawal times were of 12.75 ± 0.96, 11.79 ± 0.89, 11.32 ± 1.04, and 13.85 ± 0.72 s for saline, spadin, analog 3, and analog 8, respectively (Fig. 8a).

Because both analogs displayed the same properties and the same efficacy in behavioral tests, we decided to focalize on analog 3. This choice was supported by the fact that analog 3 is the retro-inverso of spadin and consequently shorter than analog 8. Moreover, analog 3 appeared more stable in its in vivo efficacy (see Fig. 5b) than analog 8.

TREK-1 channel deletion is known to induce epilepsy (Heurteaux et al. 2004). We analyzed the effects of analog 3, a potent blocker of TREK-1, on seizures triggered by kaïnate injection (Fig. 8b). Surprisingly, we observed that analog 3  at a dose of 10 μg/kg i.v. injected had an important protective effect against epilepsy seizures triggered by kaïnate injections (25 mg/kg in a bolus of 100 μL). Only two mice among 10 injected with both kaïnate and analog 3 have reached the two less severe stages of the epilepsy seizures, immobility, and head or neck movements; no other stages of epilepsy were observed for analog 3-treated mice. At least 9 among 10 saline-injected mice have reached the two first stages and five of them died (Fig. 8b). The effect of analog 3 was dose-dependent since a dose of 1 μg/kg showed no protective effect (Fig. 8b).

### Potential side effects on cardiac repolarizing currents

It was also important to check that analog 3 as spadin is without effects on the two main repolarizing currents at the cardiac level, the fast component I_KR_ and the slow component I_KS_. These channels are important because they are responsible for the torsades de pointe which can lead to the death. One of the most important side effects of antidepressant molecules is to induce torsades de pointe. Analog 3 did not modify currents generated either by I_KR_ or I_KS_ channels expressed in HEK cells (Fig. 8c, d).

The current densities measured for I_KR_ at 0 mV at the end of first pulse were 225.14 ± 33.09 pA/pF (*n* = 5) and 224.48 ± 35.94 pA/pF (*n* = 5) in the absence or the presence of analog 3, respectively (Fig. 8c). At the same potential, tail current densities in the absence or in the presence of analog 3 were 204.59 ± 34.18 pA/pF (*n* = 5) and 212.99 ± 38.38 pA/pF (*n* = 5), respectively (Fig. 8c). I_KS_ current densities measured at 0 mV were also very close. At the end of pulses, these values were of 17.65 ± 3.84 pA/pF (*n* = 5) and 17.58 ± 4.03 pA/pF (*n* = 5) in the absence or the presence of analog 3, respectively (Fig. 8d). I_KS_ tail current densities were of 8.33 ± 1.78 pA/pF (*n* = 5) and 8.33 ± 2.06 pA/pF (*n* = 5), in the absence or in the presence of analog 3, respectively (Fig. 8d).

### Chronic treatment

To study the effects of a chronic treatment with analog 3, we used the same strategy that we used for spadin, the MedinGel formulation (Moha ou Maati et al. 2011b). Due to fact that a single subcutaneous injection is sufficient to obtain a constant and continuous controlled release of the active molecule for several weeks, this formulation offered the advantage to reduce the stress due to a daily injection.

Formulations were prepared in a way to obtain a release of 10 μg/kg/day of peptide when injected.

**Fig. 9:**
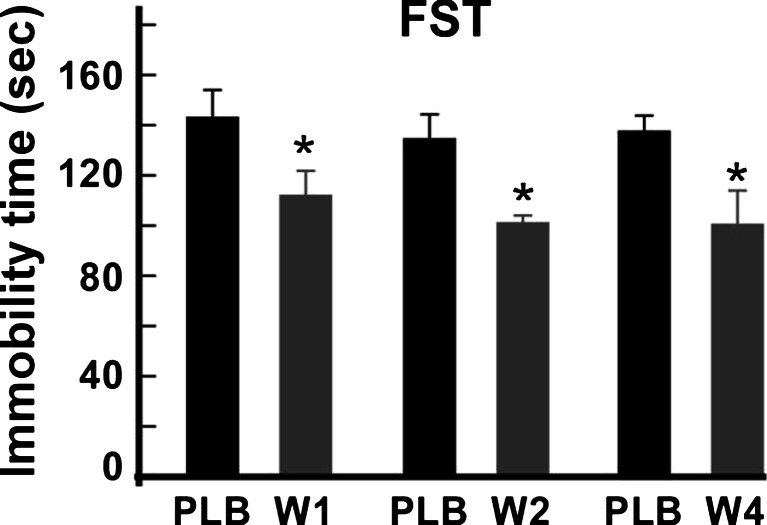
Effects of long-term treatments with spadin. Spadin–MedinGel formulation and placebo–MedinGel were subcutaneously injected in the neck of mice. Immobility times were measured in FST at 1, 2, or 4 weeks (W1, W2, W4) after injection. For each week, values obtained with formulations were compared with their corresponding placebo values by using the Mann-Whitney test. *PLB*, placebo, *p < 0.05

The efficacy of analog 3 was measured by FST after 1, 2, and 4 weeks. At each time, tested mice are naïve for the test. Mice treated with the analog 3 formulation showed a significant reduction of immobility times (Fig. 9). After 1 week, the immobility times measured were 134.40 ± 10.45 vs 112.00 ± 9.31 s (*U* = 21.5, *p* < 0.05) for the placebo-injected and analog 3 formulation-injected mice, respectively. After 2 weeks, the immobility values were 133.80 ± 11.03 vs 99.60 ± 4.92 s (*U* = 17.5, *p* < 0.05) for the placebo-injected and analog 3 formulation-injected mice, respectively. Interestingly, analog 3 released by the MedinGel formulation was still active after 4 weeks, and the corresponding values are of 137.20 ± 6.93 vs 101.10 ± 14.05 s (*U* = 20, *p* < 0.05) for the placebo-injected and analog 3 formulation-injected mice, respectively.

## Discussion

Spadin was recently identified as a new antidepressant in rodent models (Mazella et al. 2010). In this study, we identified two more efficient spadin-derived analogs. The main features of spadin as antidepressant are its rapid onset of action (4 days instead of 21 days) (Mazella et al. 2010) and the absence of side effects (Moha ou Maati et al. 2011b). Nevertheless, in  vivo Spadin half-life time measured by the FST was relatively short, around 6 h (Fig. 4). With the aim to decrease the drug intake, we decided to screen spadin analogs showing both an increased affinity for the target TREK-1 channels, which were previously identified as the target for spadin, and an increased bioavailability.

For designing these analogs, we decided to test, among others (Fig. 1), the retro-inverso (RI) peptides. For more than 20 years, it has been shown that these peptides retain their bioactivity (Chorev and Goodman 1995) and often such RI peptides display an increased bioactivity (Chorev and Goodman 1995; Taylor et al. 2000; Taylor et al. 2010). The fact that RI peptides can cross the BBB was already demonstrated for peptides involved in apoptosis of cerebral granules (Taylor et al. 2000) or for mu-opioid receptor ligands (Dooley et al. 1994).

### Electrophysiology and spadin analog screening

Among 11 analogs of spadin, the most efficient TREK-1 channel inhibition was observed with two RI analogs, analogs 3 and 8. It appeared that shorter analogs, analog 4 to 6, displayed a very low inhibition efficacy, less than 30 % when compared to the maximal inhibition. Intriguingly, analog 7 was also a bad inhibitor of TREK-1 channels, while it corresponds to the l-amino acid sequence of the analog 8 which is one of the two most potent TREK-1 channel inhibitors. It only differs by five amino acids (QDRLD) from analog 9 that displayed an inhibitory effect close to that of spadin. The importance of these amino acids in the absence of effect is partly reversed by longer peptides (see analogs 11 and 12). Analog 11 corresponds to the 44 amino acid PE released by the furin in the Golgi vesicle (Munck Petersen et al. 1999). Its reduced blocking effect on TREK-1 channels was already described (Mazella et al. 2010) but, conversely to spadin its RI-analog, analog 12 did not display a better efficacy. In summary, it appeared that better efficacies were found with RI analogs bearing the full spadin sequence such as analogs 3 and 8. Dose–response curves indicated that analog affinities were increased by a factor 5 for analog 3 and a factor 6 for analog 8. These results have allowed us to pursue the investigation with these molecules for pointing out their potential antidepressant properties and their absence of side effects on functions that are controlled by the TREK-1 channel like pain and epilepsy. We also analyzed their action on the cardiac function by measuring their effects on the two main repolarizing potassium channels in the heart.

### Spadin analogs, antidepressant effects, and neurogenesis

Both analogs 3 and 8 showed the same antidepressant properties as spadin in several behavioral tests after acute or subchronic treatments. In the FST, after an acute injection or a 4-day treatment, spadin and both analogs behaved similarly. We demonstrated that the decrease in the immobility time was not due to an excitatory effect of analogs since the spontaneous locomotor activity was similar for saline-or analog-injected mice. Analog-injected mice showed an important reduction of the latency to feed in the novelty suppressed feeding test. This test was described to be related with neoneurogenesis in the hippocampus area, a process induced by Alzheimer’s disease treatments (Malberg and Schechter 2005; Santarelli et al. 2003). Indeed as spadin did, both analogs increased the number of labelled BrdU neurons in the mouse hippocampus. We have previously shown that 80 % of BrdU-positive neurons were also double cortin positive indicating that the fate of these cells was to become neurons (Mazella et al. 2010). Our data indicated that RI-spadin analogs are not only able to bind on the spadin target the TREK-1 channel but also to trigger antidepressant spadin-like effects.

### Spadin analogs and in  vivo stability

Interestingly and as expected, these analogs presented an increased in vivo action duration. Measured by the FST, their antidepressant properties were still present 16 h after the injection. This time is about three times longer than this observed for spadin (6 h). In parallel, we showed that analog 3 is able to cross the blood–brain barrier since 30 min after the injection, a peak with a retention time corresponding to analog 3 was recovered in brain extracts. Mass spectroscopy analysis confirmed that this peak was analog 3. This peak is absent at *t* = 0, and it has completely disappeared at *t* = 60 min. The fact that the antidepressant effect of analog 3 was still measurable 16 h after injection whereas it was no more observable in the brain after 1 h could be accounted by at least two hypotheses. First, the effect is a longlasting effect and the difference between spadin and its analog could be due to the amount that reached the brain (20 times more with analog). Second, after several hours, the level of analog is too weak to be identified by HPLC analysis. A combination of both hypotheses cannot be excluded. Our data confirmed that bioactive retro-inverso peptides presented an increased bioactivity in comparison to the native structure (Chorev and Goodman 1995; Taylor et al. 2000).

### Spadin analogs and side effects

Since both analogs have the same binding and antidepressant properties, analog 3 presented the best activity/cost compromise and was investigated for potential side effects. Treating mice with analog 3 did not increase their thermal pain sensitivity, confirming data obtained with spadin (Moha ou Maati et al. 2011b). Interestingly, on kaïnate-triggered epileptic seizures, analog 3 at a dose of 1 μg/kg had no effect on the severity of seizures. But when administrated at a dose of 10 μg/kg, analog 3 showed a high degree of protection against seizures. At this dose, only two animals reached the two first level of seizure severity while nine saline-injected mice reached these levels. Treating animals with analog 3 amplified the protective effect against seizures which was glimpsed with spadin (Moha ou Maati et al. 2011b). As many drugs can induce cardiac dysfunction, among them ADs (Downes et al. 2005; Heist and Ruskin 2005), we analyzed analog 3 effects on I_KR_ and I_KS_ currents, the two main potassium channels at cardiac level, that are responsible for torsades de pointe and sudden death (Aizawa et al. 2007; Fenichel et al. 2004; Schechter et al. 2005). Analog 3 was without effects on both currents. As in the case of spadin (Moha ou Maati et al. 2011b), these results demonstrated that RI analogs of spadin did not interfere with other TREK-1-controlled pathways and did not modify the cardiac function.

### Spadin analogs and chronic treatment

Analog 3 was also used to verify that the antidepressant effect was persistent even after a chronic treatment. This experiment was performed, thanks to a MedinGel formulation that allows following a single subcutaneous injection a continuous controlled release of the peptide during several weeks (Moha ou Maati et al. 2011b). Data showed that the antidepressant effect was the same after 4 weeks and demonstrated that there was no tolerance.

## Conclusion

The three fold increase in the bioavailability associated with five- or sixfold increase in the affinity of RI-peptide for the TREK-1 channel indicated that analogs improved by a factor 15 to 18 the efficacy of spadin. Additionally, RI analogs did not induce side effects and their action was stable over the time. Taken together, these properties are very important in the aim to transform spadin or its analogs into a usable drug in human clinic. Indeed, one third of patients remain untreated because they do not correctly take their drugs. Improvements to simplify drug intake will be very helpful for these patients. In this study, we have identified a very potent spadin analog that, associated with a MedinGel formulation, could represent a great step in the spadin drug design concept for treating these untreated patients.
